# Sustained increase of spontaneous input and spike transfer in the CA3-CA1 pathway following long-term potentiation *in vivo*

**DOI:** 10.3389/fncir.2012.00071

**Published:** 2012-10-05

**Authors:** Antonio Fernández-Ruiz, Valeri A. Makarov, Oscar Herreras

**Affiliations:** ^1^Department of Systems Neuroscience, Cajal Institute-Consejo Superior de Investigaciones CientíficasMadrid, Spain; ^2^Department of Applied Mathematics, Universidad Complutense of MadridMadrid, Spain

**Keywords:** synaptic plasticity, local field potentials, long-term potentiation, independent component analysis, synfire chain, spontaneous activity, neuronal circuits

## Abstract

Long-term potentiation (LTP) is commonly used to study synaptic plasticity but the associated changes in the spontaneous activity of individual neurons or the computational properties of neural networks *in vivo* remain largely unclear. The multisynaptic origin of spontaneous spikes makes it difficult to estimate the impact of a particular potentiated input. Accordingly, we adopted an approach that isolates pathway-specific postsynaptic activity from raw local field potentials (LFPs) in the rat hippocampus in order to study the effects of LTP on ongoing spike transfer between cell pairs in the CA3-CA1 pathway. CA1 Schaffer-specific LFPs elicited by spontaneous clustered firing of CA3 pyramidal cells involved a regular succession of elementary micro-field-EPSPs (gamma-frequency) that fired spikes in CA1 units. LTP increased the amplitude but not the frequency of these ongoing excitatory quanta. Also, the proportion of Schaffer-driven spikes in both CA1 pyramidal cells and interneurons increased in a cell-specific manner only in previously connected CA3-CA1 cell pairs, i.e., when the CA3 pyramidal cell had shown pre-LTP significant correlation with firing of a CA1 unit and potentiated spike-triggered average (STA) of Schaffer LFPs following LTP. Moreover, LTP produced subtle reorganization of presynaptic CA3 cell assemblies. These findings show effective enhancement of pathway-specific ongoing activity which leads to increased spike transfer in potentiated segments of a network. They indicate that plastic phenomena induced by external protocols may intensify spontaneous information flow across specific channels as proposed in transsynaptic propagation of plasticity and synfire chain hypotheses that may be the substrate for different types of memory involving multiple brain structures.

## Introduction

The information flow between brain nuclei is made through synchronous activity in rapidly changing neuron combinations or cell assemblies (Stevens and Zador, 1998; Kumar et al., 2010). Such flow can be modulated by synaptic plasticity, a crucial mechanism in basic cognitive processes such as memory, learning, and adaptation (Martin et al., 2000; Lynch, 2004; Kandel, 2009). Specific cell assemblies in the CA1 region of the hippocampus are thought to encode sequential memories (Manns et al., 2007; Dupret et al., 2010; MacDonald et al., 2011), while the activity in the upstream CA3 region has been considered pivotal in the detection of novelty and sensory habituation by the hippocampus (Vinogradova, 2001). From the mechanistic point of view, it is difficult to relate behavioral and cognitive functions requiring long lasting changes in neural substrates with plastic phenomena induced by experimental protocols of repetitive activation in small segments of a network. In fact, the impact of these plastic changes on the spontaneous activity of single neurons remains largely unclear. There are two major difficulties to approach the experimental study of synaptic plasticity in complex neural networks *in vivo*. First, although it is accepted that information is encoded as the correlated firing of units within assemblies in a sparse and highly distributed manner (Nicolelis et al., 1997; Diesmann et al., 1999; Harris, 2005), the stability, composition, and dynamics of these assemblies are unknown. Second, spikes produced by any individual neuron may have a multisynaptic origin, complicating the correlation of ongoing changes in spike series with specific variations of incoming activity in one or another presynaptic population.

Long-term potentiation (LTP) is commonly employed in laboratory models of synaptic plasticity, in which stimulus-evoked responses are used to detect alterations in unitary or population excitability induced by the controlled activation of afferent axons (Bliss and Lømo, 1973). However, few studies have addressed the physiological correlates of LTP (i.e., the role of LTP during ongoing activity) and its effects on the dynamics of pre- and postsynaptic populations (Stevens and Zador, 1998; Dragoi et al., 2003; Whitlock et al., 2006; Yun et al., 2007). Accordingly, it is unclear whether and how potentiation of an input modifies spiking activity in a postsynaptic population, and whether such effects indicate increased efficiency in spike transfer from one relay point to the next in the network. We addressed this issue by simultaneously monitoring pairs of synaptically connected neurons and their associated excitatory stimuli, in order to sort postsynaptic spikes according to triggering inputs. This was achieved by an approach using spatially discriminating techniques (Bell and Sejnowski, 1995) that isolates CA3-elicited synaptic events from CA1 local field potentials (LFPs) as a means to identify spontaneous postsynaptic spikes in CA1 units related to a specific input (Fernández-Ruiz et al., 2012).

We previously demonstrated that some of the separated synaptic sources contributing to hippocampal LFPs are pathway-specific (Korovaichuk et al., 2010; Makarov et al., 2010; Makarova et al., 2011), describing in some detail the spatio-temporal properties of the ongoing Schaffer input to CA1 *in vivo* (Fernández-Ruiz et al., 2012). The low firing rate and functional clustering of CA3 pyramidal cells (Thompson and Best, 1989; Takahashi et al., 2010; Kimura et al., 2011) permit elementary synaptic events to be identified in Schaffer-specific LFPs (i.e., ongoing field EPSPs), which we term micro-field-EPSPs or μ-fEPSPs (Fernández-Ruiz et al., 2012). In the former study, we showed that μ-fEPSPs act as quantal excitatory packages elicited by synchronous firing of functional assemblies of presynaptic CA3 pyramidal units and that some of these μ-fEPSPs can provoke the firing of CA1 pyramidal cells and interneurons in the absence of additional concurrent inputs.

Ongoing pathway-specific synaptic activity permits a monosynaptic relationship to be established between spikes emitted by the units of presynaptic CA3 assemblies and those fired in postsynaptic CA1 units. Thus, in anesthetized rats we can quantify ongoing changes in the Schaffer input to CA1 following LTP, and determine how pairs of pre- and postsynaptic neurons modify spike transfer compared to the population. We found that the ongoing Schaffer excitation and the share of postsynaptic spikes fired by Schaffer input specifically in CA1 units increases after LTP without significant change of the mean firing rate. A re-organization of the presynaptic cell assemblies synchronously firing to elicit CA1 spikes was also found. Thus, the results provide first time evidence for pathway-specific ongoing plasticity and its impact on spontaneous network activity. Plasticity presents itself as an increased spike transfer between nuclei connected by specific potentiated channels. These observations complement and extend our understanding of classic LTP elicited by evoked stimuli; they show the ongoing correlates of LTP and support the view of synfire chains (Abeles, 1991) as a prominent mechanism for information transfer in neural networks.

## Results

### Schaffer-specific LFPs reflect the ongoing dynamics of the CA3 input to CA1

**Figure 1:**
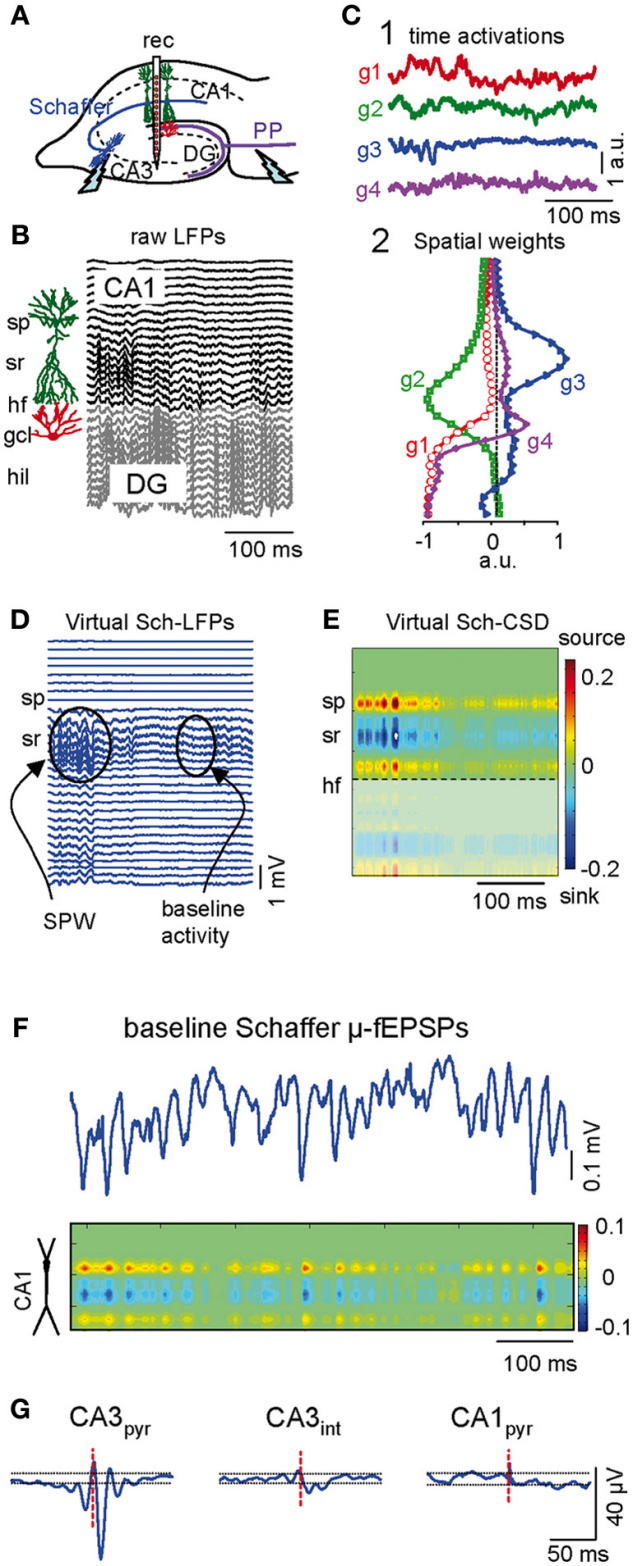
**Separation and characterization of Schaffer-specific μ-fEPSP events from raw LFPs. (A)** Schematic illustration of the electrode arrangement. *Rec*, recording. **(B)** Linear recordings of raw LFPs across the CA1 and CA3/DG fields. *Sp*, stratum piramidale; *sr*, stratum radiatum; *hf*, hippocampal fissure; *gcl*, granule cell layer; *hil*, hilus. **(C)** The independent component analysis extracts four significant LFP-generators with distinct activation **(1)** and spatial distribution profiles **(2)**: two in the CA1 region (g2 and g3) and two in the DG (g1 and g4). **(D)** Reconstructed Schaffer-specific LFPs corresponding to g3. This generator captures sharp-wave events (SPW) that stand out from the baseline activity. **(E)** Current source density (CSD) of virtual Schaffer-specific LFPs renders a unique spatial distribution of inward (blue) and outward currents (yellow-red) for spatially coherent membrane events (**C, D**, and **E** correspond to the same LFP segment shown in **B**). **(F)** Temporal extension of the baseline activity in the Schaffer generator and its CSD analysis. Note the discrete regular μ-fEPSP events (short wavelets). **(G)** Spike-triggered averages (STA) of the Schaffer-specific LFPs in the CA1 were obtained from the spikes of individual neurons (red dashed line marks spike time 0 for LFP averaging). Only presynaptic CA3 pyramidal cells (*CA*3_*pyr*_), but not the CA3 interneurons (*CA*3_*int*_) nor CA1 pyramids (*CA*1_*pyr*_), rendered STAs with significant amplitude and features analogous to stimulus-evoked Schaffer fEPSPs.

We recorded LFPs and units during irregular activity (i.e., non-theta oscillation) using multisite linear probes that spanned the CA1 and CA3 fields of the rat hippocampus (Figure 1A). Ongoing LFPs are produced by postsynaptic transmembrane currents in principal cells elicited by spontaneous synaptic inputs, therefore, they contain a time varying contribution of different sources (Elul, 1972). The CA1 region of the hippocampus has two anatomical advantages facilitating the separation and identification of presynaptic contributions, including the palisade arrangement of the main type of principal neurons (pyramidal cells) and the stratified arrangement of some of their inputs in specific dendritic domains. These anatomical features make hippocampal LFP profiles recorded right in the location of active neurons (as opposed to remote sources) particularly well suited for application of Independent Component Analysis (ICA) (Bell and Sejnowski, 1995) to separate their mixed components (here termed as LFP generators) based on their selective spatial contribution and independent temporal activation (see “Materials and Methods”).

LFP generators can be considered as dual entities, formed by one homogenous presynaptic population and the subcellular domain to which their axons project onto target neurons. Each is characterized by a constant spatial distribution (i.e., the joint curve of voltage weights for each electrode) and a temporal activation varying for different LFP segments (Korovaichuk et al., 2010; Makarova et al., 2011). The cross-animal stability, pathway specificity, and the quantitative properties of these LFP-generators have been verified previously (Korovaichuk et al., 2010; Makarova et al., 2011). Representative segments of LFPs (Figure 1B) were composed of four principal LFP-generators (g1–g4, Figure 1C). The CA3 population input to ipsilateral CA1 pyramidal cells *via* Schaffer collaterals (or Schaffer LFP-generator, g3) had an easily recognizable spatial profile with a typical hump in the stratum radiatum of the apical dendrites that closely matched the spatial profile for stimulus-evoked Schaffer fEPSPs. Subthreshold evoked fEPSPs and spontaneous sharp-wave events (SPW) were also collected exclusively into the Schafer LFP component that identified unambiguously the pathway specificity of this LFP-generator (Fernández-Ruiz et al., 2012).

Virtual LFPs produced by a single LFP-generator can be reconstructed by multiplying the specific activity (temporal activation) by the corresponding curve of spatial weights (Korovaichuk et al., 2010). Figure 1D shows virtual LFPs for the Schaffer generator (g3) containing a recognizable SPW and baseline activity. The spatial distribution of transmembrane current along the anatomy of CA1 pyramidal cells obtained by current-source density (CSD) analysis of these virtual Schaffer LFPs (Figure 1E) revealed a clean spatial distribution of current sinks (excitatory currents) in the stratum radiatum, flanked by passive sources. Such spatial distribution perfectly matched the known distributions of Schaffer-evoked field potentials (Herreras, 1990; Korovaichuk et al., 2010). On closer inspection, the baseline activity (Figure 1F) revealed a regular succession of wavelets or μ-fEPSPs at the gamma frequency (45.2 ± 1.5 Hz, estimated in autocorrelation functions; *n* = 6 animals). Moreover, the CSD distribution of μ-fEPSPs was identical to that of larger SPW events and CA3-evoked field potentials (Figures 1E,F).

The presynaptic origin of the Schaffer LFP-generator was further assessed by correlating its temporal dynamics with the firing of CA3 pyramidal cell units. Spike trains from pyramidal cells in the somatic layers of CA3 and CA1, and of putative interneurons, were isolated and classified according to their electrophysiological properties (“Materials and Methods”). Representative examples of spike-triggered averages (STAs) of the Schaffer LFP-generator were constructed for three cell types by averaging CA1 Schaffer LFPs over spikes of single cells (Figure 1G). Only CA3 pyramidal cells rendered statistically significant STAs of CA1 Schaffer-LFPs that were similar to the evoked fEPSPs, even in terms of the spatial profile and the location of the inward/outward currents along the main axis of CA1 pyramidal cells (latency, 12.1 ± 0.6 ms; amplitude, 50 ± 6 μV; duration, 17.4 ± 0.4 ms; *n* = 67 CA3 pyramidal cells in 16 animals). These results indicate that the time course of the Schaffer LFP-generator (the sequence of μ-fEPSPs) reflects the envelope of CA1 postsynaptic currents specifically produced by the firing of presynaptic CA3 pyramidal cells.

### LTP enhances ongoing CA1 excitation by CA3 input

**Figure 2:**
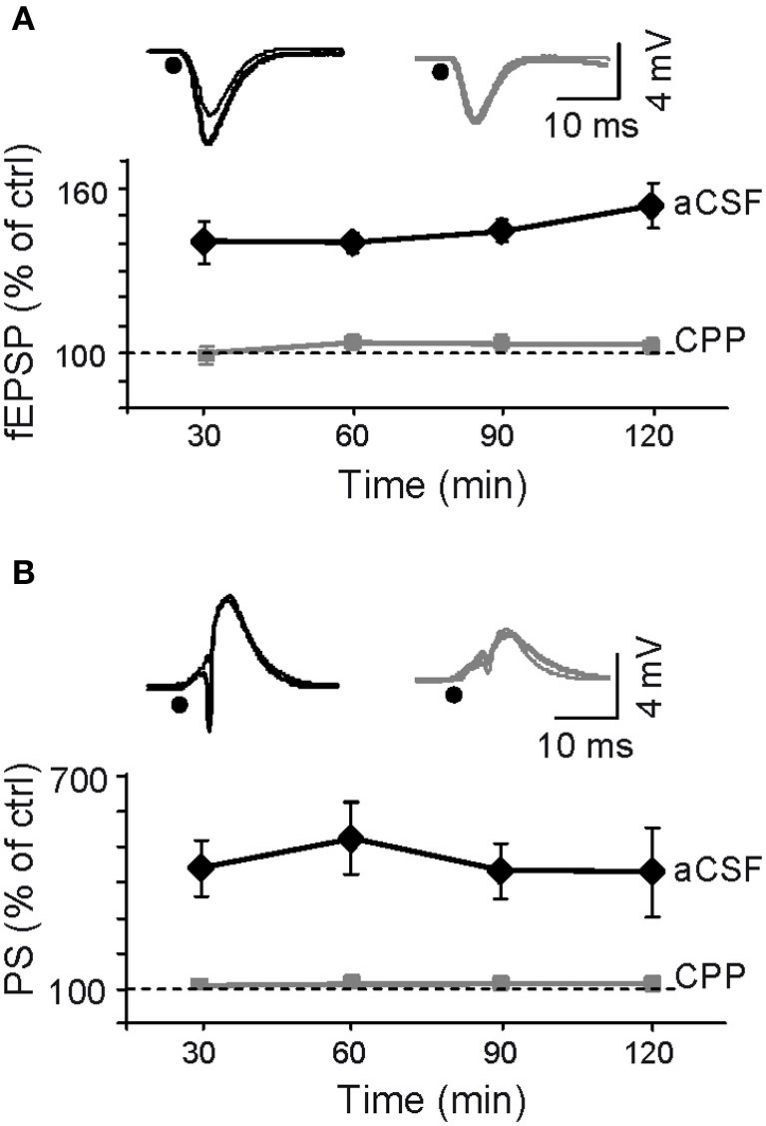
**Assessment of LTP induction by Schaffer-evoked field potentials.** LTP was induced by a burst stimulation protocol (BS: see “Materials and Methods”). **(A,B)** Initial fEPSP slope and the amplitude of the population spike (PS). Sample traces shown above the plots correspond to control (thin) and 1 h post-BS (thick) in representative experiments following prior application of aCSF (black) or CPP (gray), respectively, (black dots mark the time of stimulation). LTP of Schaffer-evoked responses was blocked by injection of the NMDA receptor's blocker in the vicinity of the probe at recording site in the stratum radiatum.

We analyzed how inducing LTP by burst stimulation (BS) of the ipsilateral CA3 affected spontaneous activity in the CA3-CA1 pathway. The successful induction of LTP was verified by the augmented amplitude of the evoked fEPSP slope and population spike (PS) (Figure 2) recorded in the stratum radiatum and pyramidale, respectively. The results in Figure 2 correspond to normalized changes (in % of pre-BS value) of *n* = 6 experiments (values are mean ± sem). The fEPSP slope increased by 145 ± 4% of control (*p* < 0.01, Student's paired *t*-test) at 1 h post-BS and local injection of ACSF, while the increase was impaired after local administration of the Glutamate receptor antagonist of the NMDA type 3-((±)-2-carboxypiperazine-4-yl)-propyl-1-phosphonic acid (CPP) delivered before BS to the CA1 stratum radiatum (102 ± 2%; *p* > 0.05; *n* = 4 animals) (gray *vs.* black traces in Figure 2) confirming the well-known NMDA-receptor dependence of LTP in the Schaffer pathway (Harris et al., 1984). These effects of BS on evoked responses were stable at least for 2 h.

We first estimated whether LTP had any global effect on the raw LFP activity of the CA1 by computing the spectral power of 10 min epochs recorded within the 30 min prior to BS and 1 h after BS (*n* = 6 animals). A moderate but significant increase (*p* < 0.05; Student's paired *t*-test) was found both for wide-band analysis (0.5–300 Hz, 126 ± 11% of control power) or specifically in the 30–100 Hz gamma-band (129 ± 14%).

**Figure 3:**
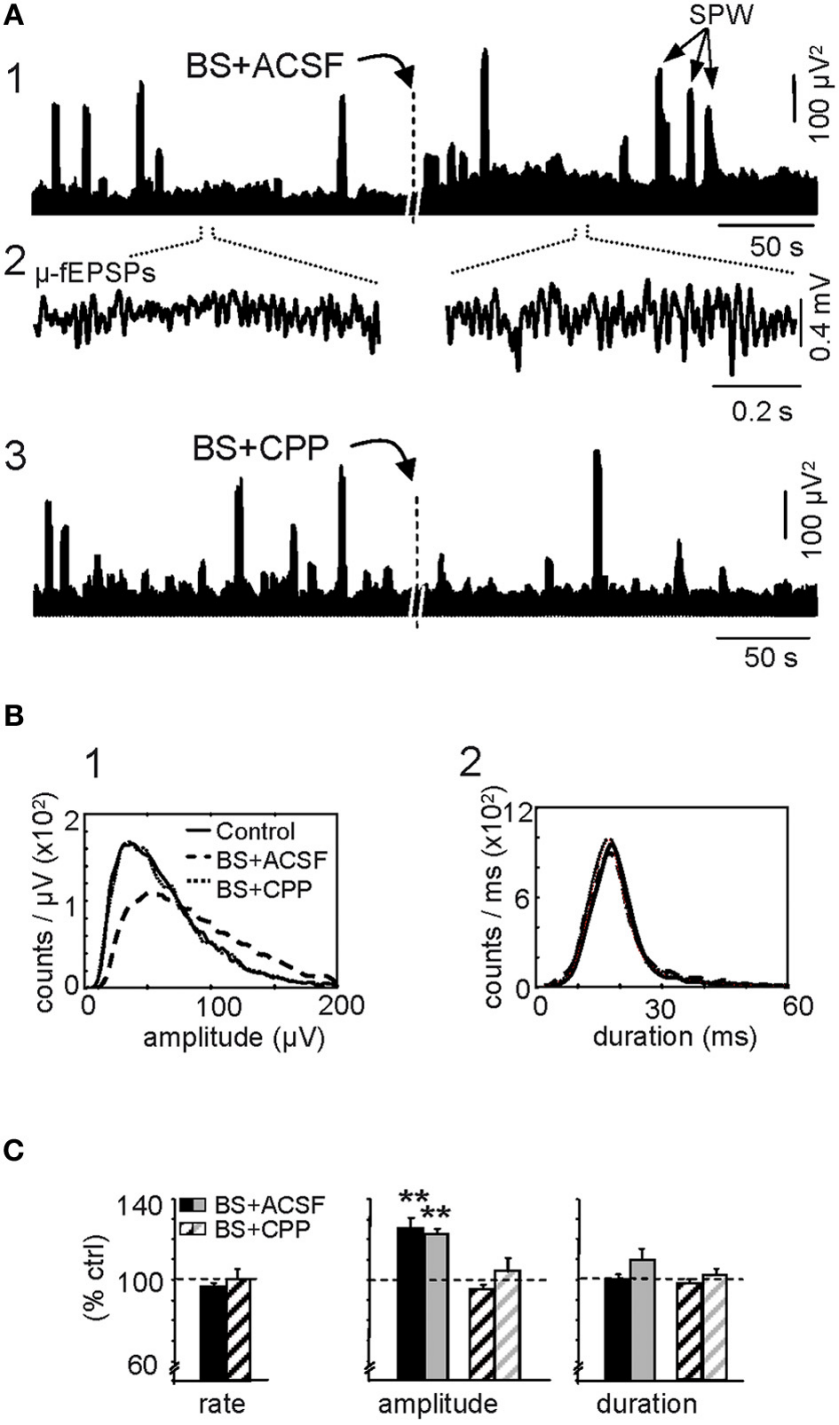
**Augmentation of ongoing Schaffer-CA1 input following LTP.** LTP is expressed as a sustained increase in the amplitude of spontaneous μ-fEPSP events during irregular (non-theta) LFPs. **(A)** The burst stimulation (BS) protocol applied to the ipsilateral CA3 produced a sustained population increase in the power of the reconstructed Schaffer LFPs (the plots illustrate two short epochs taken 10 min before and 1 h after BS, analyzed together by ICA) **(1)**. The dashes in the middle marked ~1 h period lag, at the beginning of which the BS was applied. Note the larger amplitude of elementary μ-fEPSPs in baseline activity **(2)**. The BS effect was blocked by prior local application of the NMDA-receptor antagonist CPP in the stratum radiatum of CA1 **(3)**. Large bouts of activity correspond to sharp-wave (SPW) events. **(B)** Distribution of the amplitude (left panel) and duration of μ-fEPSPs (right panel). Data correspond to 10 min epochs each for baseline (control), BS plus vehicle (ACSF) and BS in presence of CPP. **(C)** Cross-animal quantification of the effect of BS on the rate, amplitude and duration of μ-fEPSPs in the absence (solid bars) and presence of CPP (dashed bars; black and gray colors code for maximum and median values of the distribution, respectively: mean ± sem, *n* = 6 animals, ^**^p < 0.01, Student's paired *t*-test).

Next we analyzed the effect of LTP-inducing stimulation specifically on the Schaffer activity. First we examined the overall population activity as measured by the time-envelope of the power of the Schaffer generator baseline evaluated on the same epochs as above. BS induced a significant and stable increase in the mean power (173 ± 15% of control value; *p* < 0.005, Student's paired *t*-test), indicating effective potentiation of the spontaneous synaptic activity of the CA3 onto CA1. A representative example demonstrating the temporal evolution of the Schaffer LFP-generator during spontaneous activity under control conditions and after BS is shown in Figure 3A1 (note that control and treatment epochs were analyzed by the ICA as a single continuous epoch; see “Materials and Methods”). Administration of CPP through a recording glass pipette (see “Materials and Methods”) prior to BS protocol prevented these changes (101 ± 4% of pre-BS value; *p* > 0.05) (Figure 3A3).

A close examination revealed notable changes in the elementary μ-fEPSPs that constituted the baseline of Schaffer LFPs (Figure 3A2). These were sorted using a wavelet transform with the Haar mother wavelet (“Materials and Methods”). The amplitude and the duration of the Schaffer μ-fEPSP events were extracted and their distributions are shown for a representative experiment in Figure 3B (BS was applied in the presence of CPP and 2 h later the same protocol was repeated once the effect of the drug has gone by diffusion in the tissue) while the results of all 6 experiments are shown in Figure 3C. Following BS, we observed no significant change in the rate (95.2 ± 1.4% of pre-BS value: 45.2 ± 1.5 Hz) or duration (mean: 100 ± 2%; maximum value: 109 ± 6%) of μ-fEPSPs, although a significant increase in amplitude was detected (mean: 126 ± 5%; maximun: 123 ± 2%; *p* < 0.005, Student's paired *t*-test). The effect of BS on the μ-fEPSP mean amplitude was also blocked by CPP (Figures 3A3,B, respectively, *p* > 0.05 *t*-test: ACSF, *n* = 6; CPP, *n* = 4). Thus, the enhanced power of the Schaffer LFP-generator is due to an increase in the amplitude (but not the rate) of the contributing elementary μ-fEPSPs, consistent with previous reports of the effects of LTP on standard evoked fEPSPs (Figure 2).

These results indicated a global potentiation of Schaffer LFPs following LTP that was much stronger than that of raw LFPs, highlighting the usefulness of isolating pathway-specific LFPs.

### LTP induces sustained changes in pre- and post-synaptic spike activity, and increases the efficiency of CA3-CA1 spike transfer

**Figure 4:**
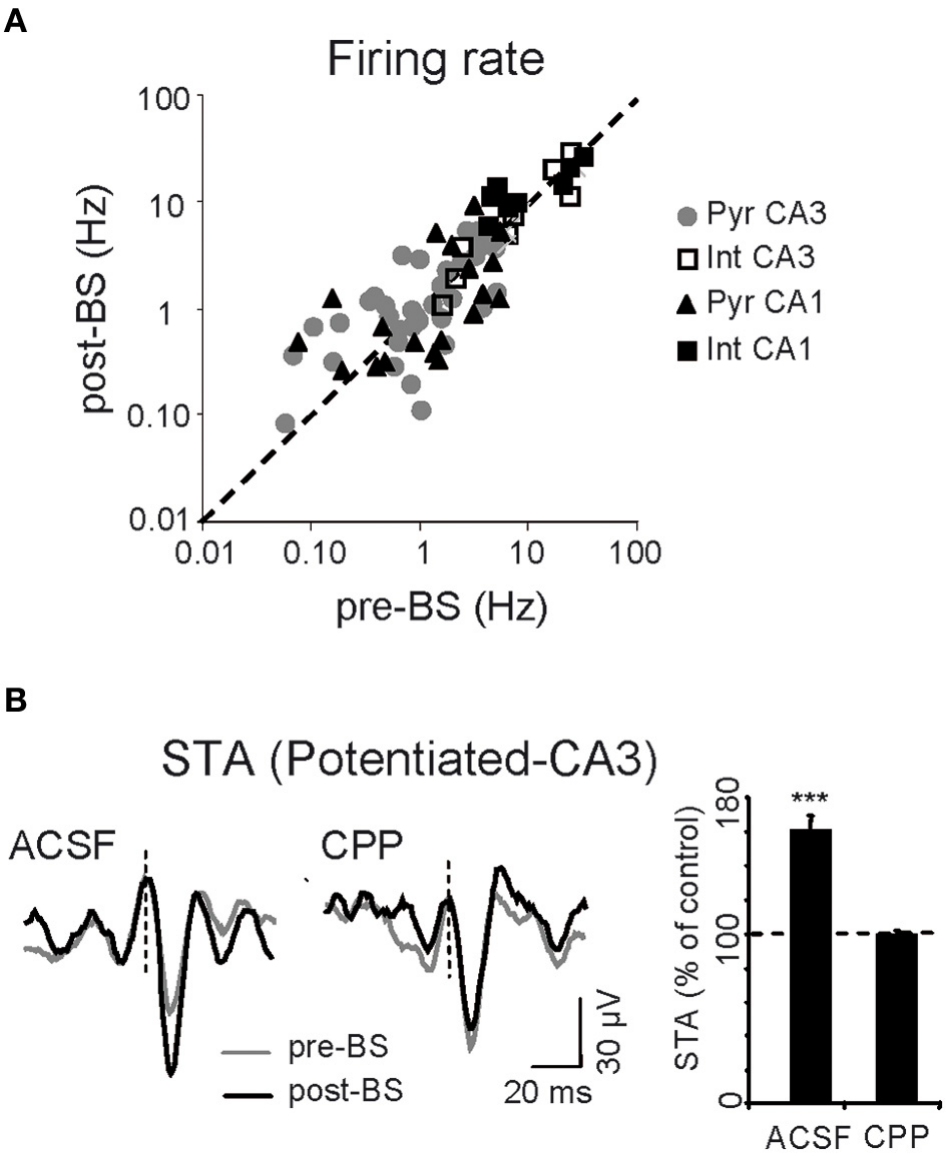
**Cell-specific effects of LTP. (A)** Mean spontaneous firing rate of CA1 and CA3 cells before and after BS. Each symbol corresponds to a different neuron (*n* = 6 animals). **(B)** Representative sample traces (left) and pooled results of all six experiments (right histogram) for CA3 spike-triggered averages (STA) of Schaffer LFPs before (gray) and after BS (black), following local administration of vehicle (ACSF) or CPP in the CA1 stratum radiatum. The histogram corresponds to the subgroup of CA3 pyramidal cells that showed STA potentiation. Vertical dashed lines indicate the spike time (zero) for LFP averaging (^***^p < 0.001, Student's paired *t*-test).

Having shown that LTP induction enhances the ongoing CA3 excitatory input to CA1 as reflected by Schaffer LFPs, we explored whether and how this change affected the firing of individual units in both pre- and postsynaptic areas. Intuitively we might expect that the spontaneous firing rate in at least the postsynaptic region would be increased after LTP. The firing rates of pyramidal cells and interneurons were estimated over 25 min epochs in control conditions and 1 h after BS (*n* = 6 animals; Figure 4A): (1) CA3 pyramidal cells, 1.6 ± 0.2 Hz *vs.* 1.5 ± 0.2 Hz (*n* = 36); (2) CA3 putative interneurons, 10.4 ± 3.4 Hz *vs.* 9.2 ± 3.0 Hz (*n* = 10); (3) CA1 pyramidal units, 1.9 ± 0.4 Hz *vs.* 1.5 ± 0.4 Hz (*n* = 20); (4) CA1 putative interneurons, 12.3 ± 4.0 Hz *vs.* 12.8 ± 2.2 Hz (*n* = 9). Thus, BS did not appear to significantly modify the firing rate of either subclass of neurons over long periods (*p* > 0.05, *t*-test). The resulting population invariance was consistent with previous reports (Martin and Shapiro, 2000; Dragoi et al., 2003), although some individual units exhibited up to 10-fold variation in the firing rate following LTP (note the log scale in Figure 4A), indicating that cell-specific changes were balanced at the population level. Additional observations guided us to an explanation for this apparent paradox. We noted that only some CA3 pyramidal cells potentiated the STA of Schaffer LFPs in the CA1 region (19 out of 28 cells, mean increase = 159 ± 7%: Figure 4B). This cell-specific increase in STA did not occur following CPP injection (*p* > 0.05, Student's paired *t*-test; *n* = 4 animals) and thus only a fraction of active presynaptic units appeared to be involved in the potentiation observed at the postsynaptic site.

**Figure 5:**
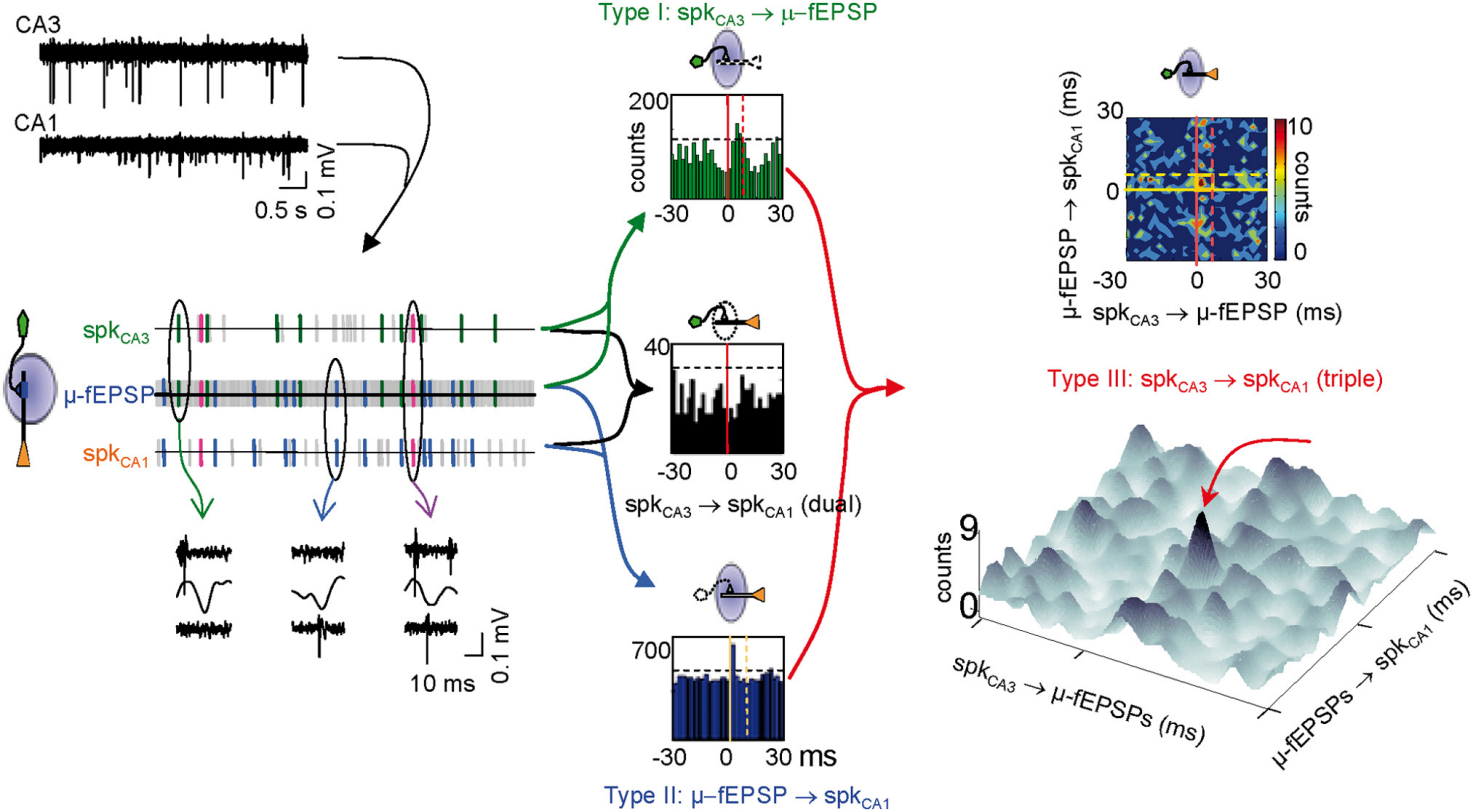
**Identification of monosynaptically connected cell pairs in the CA3-CA1 network.** Using the excitatory quanta composing the baseline activity of Schaffer-LFPs (μ-fEPSPs) allows discriminating synaptically connected CA3 and CA1 units. The upper left traces correspond to filtered simultaneous multiunit recordings in the stratum pyramidale of the CA3 and CA1 regions. Following spike sorting, spike trains of pairs of pre- and postsynaptic units were correlated with point processes of concurrent Schaffer μ-fEPSPs obtained from LFPs. Plausible monosynaptic coincidences are color coded as follows: Type I, green (in-cluster firings); Type II, blue (Schaffer spikes); Type III, magenta (Spike transfer efficiency). An example of raw data for each case is shown below (ovals). Horizontal dashed lines in dual histograms mark the significance level (α = 0.05, Surrogate test). Counts contained within the periods delimited by vertical lines in dual cross-correlations of Type I (green histogram) and II (blue histogram) are candidates for monosynaptic relations. Significant correlations in dual histograms do not ensure significant CA3 to CA1 spike transfer when examined by standard cross-correlation of spike trains in dual histograms (e.g., black histogram). However, the sorting of spikes in the respective trains of the CA3 and CA1 units by a common temporal locking to μ-fEPSPs (Type III coincidences plotted in bidimensional densitograms: upper right panel) reveals few spikes within a monosynaptic time window (golden square in densitogram) at higher than chance density. In densitograms, zero means the spike and μ-fEPSP are coincident. Positive and negative numbers on the X-axis mean the nearest CA3 spike occurred before or after the μ-fEPSP, respectively. Positive and negative numbers on the Y-axis mean the nearest CA1 spike occurred after or before the μ-fEPSP, respectively. The pseudo 3D plot below allows a better visualization of significant triple coincidences (peak marked by red arrow). Note that the level of the background may have no physiological meaning except for spurious or subsidiary relations.

We next analyzed the alterations in the dynamics of pre- and postsynaptic units (see Figure 5 for a scheme of all temporal relations in the synaptic chain). We previously reported that each μ-fEPSP is elicited by a functional cluster of CA3 pyramidal cells to which individual pyramidal neurons contribute in a variable manner (i.e., a fraction of spikes in each individual CA3 pyramidal cell is coupled temporally with μ-fEPSPs, so called *in-cluster* firings; Figure 5, green lines in left panel). All pyramidal CA3 units examined contribute to the generation of μ-fEPSPs in the CA1 (Type I relationship), with a mean 23% of their spikes fired in synchrony with spikes of other cells forming a functional assembly, which jointly elicit μ-fEPSPs (*in-cluster* spikes). Moreover, a fraction of the spikes in each CA1 unit are temporally locked to μ-fEPSPs, known as Schaffer spikes (Type II relationship) (Figure 5, blue lines in left panel). Excitatory Schaffer input contributes decisively to 11% of the spikes in 20 out of 29 (70%) of CA1 pyramidal cells (Fernández-Ruiz et al., 2012). Thus, we searched for dual and triple coincidences of these three elements in the synaptic chain using time windows appropriate to ensure monosynaptic excitation and we determined their significance using a surrogate test (see Figure 5 central panel and “Materials and Methods”).

The test for triple coincidences (see Figure 5 right panels and “Materials and Methods”) reveals the fraction of μ-fEPSPs that were simultaneously time-locked to one pre- and one postsynaptic spike within defined time windows. Thus, pairs of pre- and postsynaptic spikes are selected by their time-locking to a common μ-fEPSP, unveiling temporal correlations that would normally remain buried in standard dual correlations of spike trains (as in the black histogram in Figure 5). The triple correlations are built by cross-correlating presynaptic in-cluster firings to postsynaptic Schaffer-spikes. These were plotted as two-and tridimensional densitograms. Since distant pre- and postsynaptic spikes may only have subsidiary relations of complex physiological interpretation, we set a monosynaptic time window of 6 × 8 ms (termed window *a*: golden box in contour densitogram of Figure 5) in which the timing of pre- and postsynaptic spikes to the μ-fEPSP may indicate direct connection between the cells, i.e., the presynaptic unit contributed to a μ-fEPSP, which in turn fired a spike in a postsynaptic unit. If the density of triple events within this window is higher than outside (we set for reference a 30 × 30 ms time window, termed window *b*) the chances are that they are causally related, i.e., that a presynaptic spike contributed significantly to the firing in the postsynaptic unit (spike transfer). We set an *a/b* ratio or spike transfer rate index higher than 1.2 (density is 20% higher in the monosynaptic window: see “Materials and Methods”) as a lower limit to establish effective connections. In these cases a high density spot stands out from background. Positively correlated pairs are better appreciated in pseudo-3D plots (Figure 5).

#### Upstream (presynaptic) changes

**Figure 6:**
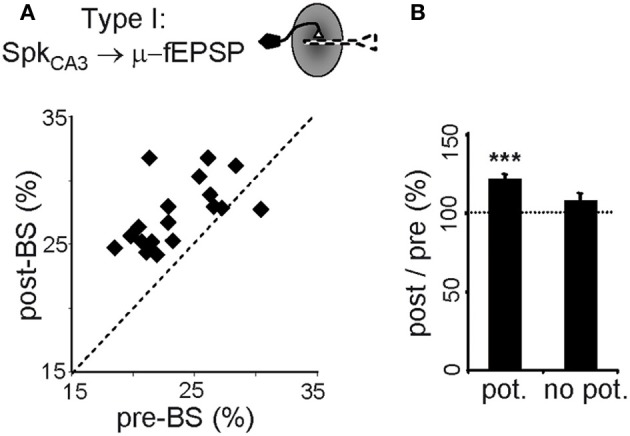
**Persistent upstream changes in the CA3-CA1 pathway. (A)** Correlation between percentage of spikes of potentiated CA3 pyramidal cells monosynaptically related to μ-fEPSPs (Type I relationship) before and after BS. Note that the values fall above the midline. **(B)** Only those CA3 pyramidal cells exhibiting potentiated spike-triggered averages in CA1 (*pot*.) increase the post/pre BS *in-cluster* presynaptic firing. Data obtained from 10-min epochs taken before and 1 h after BS (mean ± sem, *n* = 6 animals; ^***^p < 0.001, Student's paired *t*-test).

In CA3 pyramidal neurons, BS significantly increased the proportion of spikes monosynaptically associated with μ-fEPSPs (119 ± 3% of controls, *p* < 0.005, Student's *t*-test; Figure 6), specifically in cells exhibiting a potentiated STA (Figure 6B). Thus, LTP increased the percentage of spikes fired by CA3 pyramidal cells that contributed to μ-fEPSPs, indicating more frequent recruitment of presynaptic units into effective functional assemblies. Also, we checked for changes in the internal dynamics of CA3 pyramidal cells by cross-correlating spike trains of simultaneously recorded units before and after BS. Out of 36 cells in all animals we were able to examine 90 cell pairs, of which 26 (29%) increased significantly their spike-to-spike synchronization after BS (time-window of ± 2 ms), whereas 13 pairs (14%) reduced it (level of significance was ± 20% of pre-BS value). The number of cells is however too small to find statistically significant relations between the pre-BS presence or absence of synchrony and the post-BS groups. Taken together, these two observations suggest that some cells may drop from a functional assembly while others may incorporate into a new or a different one following LTP induction.

#### Downstream (postsynaptic) changes

**Figure 7:**
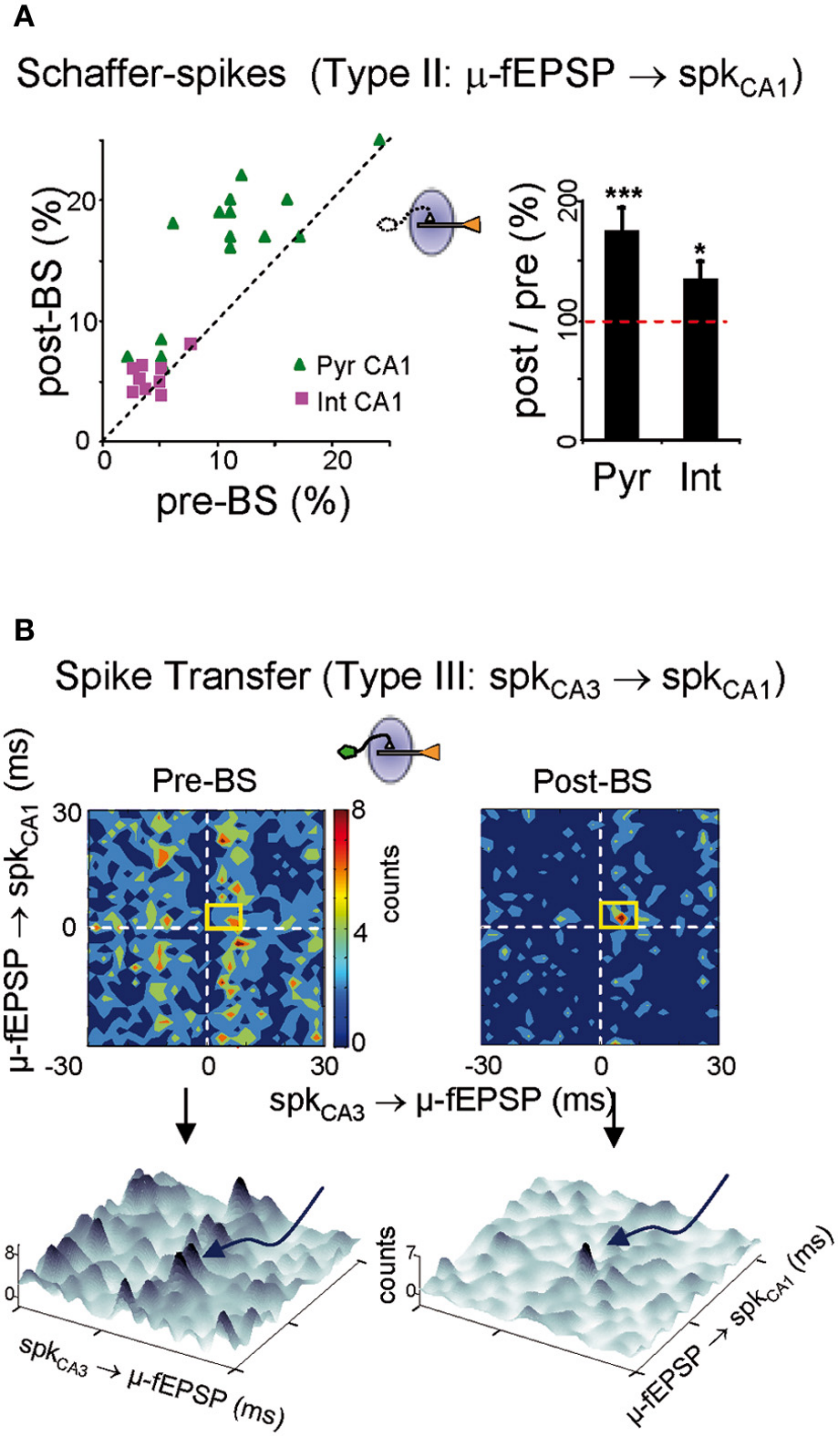
**Persistent downstream changes in the CA3-CA1 pathway. (A)** Postsynaptic CA1 units increased their share of Schaffer-driven spikes in 25 min temporal series 1 h after BS. This increment was greater in pyramidal cells (green triangles; *Pyr CA1*) than in interneurons (magenta squares; *Int CA1*: ^***^p < 0.001, ^*^p < 0.05, Student's *t*-test; *n* = 6 animals). **(B)** BS increased the spike transfer efficiency and cell-to-population salience of CA3-CA1 pyramidal cells pairs. Color-coded contour densitograms and the corresponding pseudo 3D representations plot the density of the time coincidences of CA3 spikes locked monosynaptically to CA1 μ-fEPSPs against those of CA1 spikes monosynaptically locked to μ-fEPSPs, for one representative experiment (time epochs of 10 min before and 1 h after BS). The monosynaptic window (6 × 8 ms) indicated by the yellow squares captures successful monosynaptic spike transfer between the two cells. In 3D plots these are grouped in the peak marked by curved arrow in pre-BS and the main peak in post-BS. Salience is only increased in pre-post synaptic cell pairs in which the CA3 unit exhibited potentiated STA of the CA1 Schaffer LFP.

We analyzed the changes in the rate of Schaffer-driven spikes in CA1 units (i.e., the proportion of spikes time-locked to μ-fEPSPs within a monosynaptic time window: 2–6 ms). After BS, the percentage of Schaffer-driven spikes increased significantly in both pyramidal cells (174 ± 20 % of pre-BS value; *p* < 0.001, Student's *t*-test; *n* = 15; Figure 7A green triangles) and interneurons (134 ± 16%; *p* < 0.05, *t*-test; *n* = 9; magenta squares). This result contrasted to the comparison of unsorted spike trains that did not show any increase in the population firing rate (Figure 4A). Thus, the extraction of Schaffer-driven spikes let us visualize the pathway-specific effect of LTP induction on the spike production by postsynaptic CA1 cells that otherwise would remain hidden.

#### Changes in CA3-CA1 spike transfer efficiency

Finally, we analyzed the ability of spikes in CA3 units/assemblies to generate spikes in CA1, without the concurrence of other inputs. This was achieved by evaluating triple correlations: CA3 spikes/μ-fEPSPs/CA1 spikes or *a/b* ratios as indicated above. Densitograms were computed over 10 min epochs before and 1 h after BS. Cell pairs were classified on the basis of whether the STAs in the presynaptic CA3 unit were potentiated (19 out of 28) or not (9 out of 28: Figure 4B), and the results were crosschecked with all the simultaneously recorded postsynaptic CA1 units. In the potentiated group (19 CA3 units) we obtained a total of 68 CA3-CA1 pairs of pyramidal units. Of these, only 12 pairs exhibited efficient postsynaptic driving in control (i.e., the *a*/*b* ratio was larger than 1.2), and all of them increased the *a*/*b* ratio following LTP induction (a/b ratio = 133 ± 9% of pre-BS value; *p* < 0.05, Student's *t*-test) as evident by the higher density in the monosynaptic window in the example of Figure 7B. A reduced background was often found after BS (3D plots in Figure 7B). Globally, the results indicate enhanced synaptic transmission and spike transfer between these cells. Both, the augmented density in the monosynaptic window and the reduced background contributed to the increased cell-to-population salience of potentiated CA3-CA1 cell pairs after BS, as captured by the a/b ratio. Interestingly, in the non potentiated group (9 CA3 units) no changes were observed (a/b ratio = 106 ± 9% of pre-BS value, estimated from eight functionally connected pairs out of 32 CA3-CA1 pairs; *p* > 0.05, Student's *t*-test). These results demonstrate that LTP exerts a cell-specific effect on ongoing synaptic transmission, affecting both pyramidal cell and interneuron populations, and with a measurable impact on the output of the target region.

## Discussion

The multiple combinations of concurrent synaptic inputs that initiate outgoing spikes represent a major obstacle to analyze the informational content of temporal spike series recorded in a given brain region. In the present study, we isolate one such input (Schaffer to CA1) and studied its plastic modulation following the induction of LTP. The retrieval of elementary Schaffer-specific μ-fEPSPs in the CA1 region allowed us to link the spiking activity of CA1 and CA3 pyramidal neurons, and to identify monosynaptically connected cell pairs (Fernández-Ruiz et al., 2012). Thus, Schaffer-driven spikes in a postsynaptic CA1 unit could be sorted and the spike transfer in this section of hippocampal circuit quantified. BS of the ipsilateral CA3 region produced a sustained increase in the Schaffer excitatory drive to the CA1 by increasing the amplitude of elementary μ-fEPSPs, without altering the rate or duration of events. In turn, this response augmented the proportion of Schaffer-driven spikes in the spontaneous output of CA1 units without changing the global population firing rate. To our knowledge, this is the first evidence that LTP induction produces a pathway-specific enhancement of ongoing activity that is effectively propagated to subsequent relays of the network. Taken together, our results suggest a sustained cell-pair-specific increase in spike transfer along potentiated sections of the hippocampal circuit that are reminiscent of learning-induced memory traces.

The success and functionality of LTP-induction protocols are normally evaluated by estimating the firing probability of single cells or the increment of ensemble fEPSPs following test stimuli (Bliss and Lømo, 1973; Martin et al., 2000; Lynch, 2004). However, changes in evoked activity do not necessarily translate into spontaneous activity (e.g., Martin and Shapiro, 2000) since evoked responses only assess the sensitivity but not the activity of the stimulated pathway, in as much as spiking activity is the result of multiple convergent pathways. On the other hand, there have been few attempts to relate changes in LFPs associated to LTP induction (e.g., Bikbaev and Manahan-Vaughan, 2007) but changes could not be assigned to specific presynaptic populations. Evaluating the impact of one specific pathway among all the convergent inputs to a given brain area in spontaneous conditions is hard to achieve by standard experimental approaches. The reported effects of LTP protocols on spontaneous firing of hippocampal neurons are globally confusing, some increasing some decreasing, even within the same population and assay (Deadwyler et al., 1976; Kimura and Pavlides, 2000; Martin and Shapiro, 2000; Dragoi et al., 2003). Amongst possible confounding factors is the fact that external activation of axon bundles or nearby groups of cells does not reproduce the natural activation of specific groups of fibers or cell clusters in behaving animals through which correlated activity flows normally. The forced cooperativity amongst non-natural groups of individual inputs may be variably decoded and weighed by postsynaptic neurons. Besides, the output of principal cells is heavily controlled by several local inhibitory networks, and it is known that Schaffer collaterals activate CA1 interneurons some of which also undergo LTP (Maccaferri and McBain, 1996; Kullman and Lamsa, 2007), making it difficult to predict whether spike transfer will be enhanced by increased excitation of principal cells, or balanced by changes in inhibitory tone. In this work, we were able to clarify this issue as we found that BS induces the sustained increase in the excitatory input from the CA3 to CA1. Furthermore, this increase in excitation was effective in the output of all the CA1 cell types targeted, as the proportion of Schaffer-driven spikes increases in both pyramidal cells and interneurons. In agreement with previous studies (Martin and Shapiro, 2000; Dragoi et al., 2003), no change was evident in the mean population firing rate of CA1 pyramidal cells, despite visible LTP of evoked responses. However, we go further by showing that the increased proportion of Schaffer-driven spikes in these pyramidal neurons ensured that LTP projected the CA3 output beyond the CA1 to successive relay points in the network, thus exerting a greater impact than other synaptic inputs that converge on CA1 units. This mechanism is consistent with the view of plasticity in synfire chains, in which a pathway-specific origin of some spikes is required (Abeles, 1991). It also agrees with reports showing transsynaptic propagation of plasticity through and beyond hippocampal stations (Yeckel and Berger, 1990; Davis et al., 1996).

Classic features of LTP conventionally evaluated include pathway specificity and cooperativity. Since we used here μ-fEPSPs elicited by presynaptic clusters to link pre- and postsynaptic cells, the present results show that expression of ongoing LTP is cell- and cluster-specific, and dependent on pre-existing anatomo-functional (hard-wired) connections between pre-and postsynaptic units (i.e., cell pairs that show greater-than-chance pre- and postsynaptic spike-locking to μ-fEPSPs before LTP induction). Indeed, enhanced STA of CA1 Schaffer LFPs by the presynaptic CA3 unit and increased CA3-CA1 spike transfer was observed only in cell pairs in which Schaffer excitatory packages were capable of generating postsynaptic spikes in control conditions. Although we found no functional evidence for newly connected cell pairs following LTP, such possibility cannot be ruled out. We thus infer that lasting changes in spike transfer are more efficiently expressed when the activated set of cells/fibers coincides with those forming natural assemblies assorted by former experience. This result also contributes to the view that the fundamental computational entity in neural circuits is the cell assembly and that dynamic and plastic modulations of their functional connectivity underlie information encoding and storage in the network (Nicolelis et al., 1997; Harris, 2005; Fernández-Ruiz et al., 2012).

It is noteworthy that LTP increases the recruitment of individual CA3 pyramidal neurons to successful spike-generating μ-fEPSPs. Given that these neurons fire synchronously in functional assemblies (Hájos and Paulsen, 2009; Takahashi et al., 2010), which appear to be the functional units that give rise to μ-fEPSPs (Fernández-Ruiz et al., 2012), this observation suggests a functional reorganization of individual contributions to CA3 assemblies following LTP, possibly through the extensive recurrent networks in the CA3 region (Li et al., 1994). Whether these contribute to increased size of Schaffer μ-fEPSPs and spike transfer across this hippocampal segment is unclear. As we reported earlier, only a fraction of spikes emitted by a single cell contribute to μ-fEPSPs, which can be interpreted as a combinatorial mode of operation of functional assemblies in which not all cells need to contribute every spike. Thus, a functional assembly may be contributed by a different set of units from a larger pool in different instances, enabling large flexibility: for instance, individual cells may belong to multiple assemblies and reinforce their contributions to one or another according to processing demands. We have investigated the internal dynamics of these assemblies by searching for changes in spike-to-spike synchronization between pairs of simultaneously recorded CA3 units following LTP induction. Less than half the pairs examined show increased or decreased synchronization, which also points to a re-organization of CA3 functional assemblies after BS. Unfortunately, the linear arrays limit the number of units that can be recorded simultaneously and our population is too small to reliably come to a definitive conclusion. However, it should call our attention to upstream changes that may occur upon LTP protocols and could go unnoticed. For instance, LTP in the CA3 is collateral-specific, i.e., it may develop in one but not all of the postsynaptic populations targeted by CA3 axon collaterals (McNaughton and Miller, 1986), thus subtle differences in the protocol of induction may alter differentially the effects on different postsynaptic cell types and regions. LTP has been observed between CA3 pairs of neurons and evoked potentials, but the associated changes in functional connectivity amongst cell pairs were balanced at the population level (Debanne et al., 1998; Yun et al., 2007). Since the BS stimulation we use here seems not to have an impact on the gamma-patterned spontaneous output of CA3 we may suggest that the presynaptic firing cluster is not noticeably affected by probabilistic contribution of individual cells. The invariance of gamma-sequence in CA3 output after LTP indicates the preservation of local network mechanisms making up the pace, possibly the local inhibitory networks (Hájos and Paulsen, 2009).

Unfortunately, little is known about the physiological interpretation and the computational operations performed by the hippocampus during irregular LFPs (Buzsáki et al., 1983). The synchronous SPW events that populate these periods have been proposed as markers or predictors of memory performance (Dupret et al., 2010), and even as natural LTP-inducing stimuli (Buzsaki et al., 1987). One interesting hypothesis is that the potentiated Schaffer μ-fEPSPs between SPW events may express a natural Hebbian protocol akin to repetitive spike-timing-dependent plasticity (Caporale and Dan, 2008), particularly since they exhibit associated pre- and postsynaptic firing within the appropriate time window.

There is growing evidence that single experiences are sufficient for memory acquisition (Fyhn et al., 2002; Nakazawa et al., 2003; Whitlock et al., 2006), and that this form of learning involves the sustained potentiation of evoked f-EPSPs in the CA3-CA1 pathway, as occurs in repetitive learning (Gruart et al., 2006) and stimulus-induced LTP (Bliss and Lømo, 1973). The use of artificially evoked or naturally occurring synchronous activity patterns (such as SPW events) to assess synaptic plasticity (King et al., 1999) is not very informative regarding the asynchronous nature or even the sign (facilitated or depressed) of the ongoing information transfer, and of the complexity of computations that are performed in neural circuits. If sustained changes in spike transfer between specific cells and nuclei occur, they probably underlie changes in neural representations of learned information and thus, they should be further analyzed to determine their role in behavior as well. Indeed, single trial memory acquisition is essentially an ongoing activity and no doubt involves transsynaptic propagation of plasticity through multiple stations in a network. Since it is plausible that memory traces are represented by structural or activity changes in parallel chains (the multiple cell–cell connections between two nuclei), classifying spikes based on their triggering inputs will help determine whether a particular epoch of ongoing cellular activity (i.e., a spike train) constitutes an element of learned information (e.g., Hirase et al., 2001), thereby facilitating the search for alterations in the synaptic efficiency of specific pathways that would otherwise remain masked.

## Materials and methods

### Experimental procedures

Adult female Sprague–Dawley rats were anesthetized with urethane (1.2 g/kg, i.p.) and placed in a stereotaxic device. Surgical and stereotaxic procedures were performed as previously described (Canals et al., 2005; Makarova et al., 2008). A stimulating electrode was placed in the ipsilateral CA3 region for orthodromic activation of CA1. Linear multisite silicon probes (Neuronexus, Ann Arbor, MI) of 32 recording sites were used to record in 50 μm steps along the main axis of the CA1 pyramidal cell region, also spanning the DG/CA3 regions. The probes were soaked in DiI (Molecular Probes, Invitrogen, Carlsbad, CA) before insertion for postmortem evaluation of their placement in histological sections. A silver chloride wire in the neck skin served as a reference for recordings. Signals were amplified and acquired using MultiChannel System (Reutlingen, Germany) recording hardware and software (50 kHz sampling rate).

The experiments were performed in accordance with European Union guidelines (2003/65/CE) and Spanish regulations (BOE 67/8509-12, 1988) regarding the use of laboratory animals. The Research Committee of the Cajal Institute approved the experimental protocols.

### LTP induction and pharmacological study

LTP was induced by BS of the CA3 pyramidal layer (10 trains at 0.5 Hz, administering 20 square biphasic pulses (100 μs) at 200 Hz, which were repeated three times at 5 min intervals, making a total of 600 pulses). The intensity of the stimulus was adjusted to obtain 30–50% of the maximum CA1 PS (200–400 μA range). Stimuli at the same intensity were presented every 5 s and field responses were averaged over a ten minutes period prior to BS in order to obtain baseline PS values. The fEPSP baseline values were obtained in the same way but using sub-threshold pulses (70–200 μA range). The effect of BS on evoked responses was checked by test stimuli over 5-min periods in four epochs, 30, 60, 90, and 120 min after BS, respectively. LTP induction was considered successful when the initial slope of the fEPSP was augmented by at least 20% for at least 2 h.

The pharmacology of LTP was assessed by local application of the NMDA-receptor blocker CPP obtained from Tocris (Bristol, UK). We injected microdrops of drug solutions (50–100 nl) through a recording glass pipette (7–12 μm at the tip) in the vicinity of the recording linear array at the level of the CA1 stratum (stratum) radiatum through a Picospritzer (General Valve) (Canals et al., 2005). Two microdrops were injected separated by 5 min interval before the BS stimulation. As a control, similar injection protocol was made with pipettes filled with ACSF. The pipettes were also employed for extracellular recording to correctly locate the site of injection guided by characteristic Schaffer-evoked field potentials. The drugs were dissolved in ACSF to a final concentration 50 times higher than that usually employed *in vitro* (CPP: 0.5 mM). In order to use the animals as their own controls we set the following sequence: 1 h baseline recording was followed by CPP injection and BS, and 2 h later the pipette containing CPP was exchanged by another filled with ACSF, after which ACSF was injected and BS delivered again.

Wide-band LFPs (that included unitary spikes) were recorded in 25 min epochs before BS and between 1 and 2 h after BS. We chose for analysis only epochs of irregular LFP activity, i.e., theta epochs were rejected (the presence of theta was detected by wavelet spectrum and Fourier spectrum analyses). LFP segments of different epochs can be grouped together for mathematical analysis, enabling homogeneous treatment of data for separation of Schaffer-specific activity.

### Independent component and current source density analyses of LFPs

Depth profiles of LFPs in the hippocampus show laminar distribution and contain a time varying mixture of synaptic currents from multiple presynaptic origins, making difficult to detect periods contributed by only one synaptic input. Taking advantage of the spatial constancy of the electrical fields created by synaptic inputs from the same presynaptic population that make contact in a narrow dendritic domain of postsynaptic cells we applied blind source separation techniques as the ICA (Bell and Sejnowski, 1995) to separate spatially independent components in laminar LFP profiles, some of which we showed earlier are pathway-specific (Korovaichuk et al., 2010; Fernández-Ruiz et al., 2012). Thus, even if several inputs co-activate, each produces a postsynaptic potential with different spatial profile and their respective time activity is segregated into different components (Makarov et al., 2010; Makarova et al., 2011). The location of recording sites in the array relative to anatomical boundaries is assessed by the characteristic depth profile of evoked potentials (Herreras, 1990) and histological verification. Separated component thus reflect a spatio-temporal convolution of the population synaptic activity from a specific presynaptic origin.

Detailed procedures for ICA of linear profiles of LFPs have been described previously (Makarov et al., 2010). The mathematical validation and interpretation of ICA components in laminated structures is provided by realistic LFP modeling in Makarova et al. (2011). Briefly, the 32 simultaneously recorded LFP signals were represented as the weighted sum of the activities of *N* neuronal sources or LFP-generators: *u*(*t*) = ∑^*N*^_*n* = 1_*V*_*n*_*s*_*n*_(*t*), where *V*_*n*_ and *s*_*n*_(*t*) are the vector of the spatial weights and the time course of the *n*-th LFP-generator, respectively. Thus, the raw LFP observed at the *k*-th electrode tip is a linear mixture of the electrical activity of several independent LFP-generators describing transmembrane current source densities in principal cells *I*_*n*_ = −σΔ*V*_*n*_, where σ is the conductivity of the extracellular space. To perform the ICA we employed the infomax algorithm (Bell and Sejnowski, 1995) using the EEGLAB Matlab toolbox (Delorme and Makeig, 2004), which returns the activations *s*_*n*_(*t*) and spatial weights *V*_*n*_ of up to 32 LFP-generators. Only a few generators exhibited significant amplitude and spatial distribution (e.g., four in Figure 1). Once extracted from the raw LFPs, each LFP-generator can be analyzed independently by re-constructing virtual LFPs produced by a single generator: *u*_*j*_(*t*) = *V*_*j*_*sj*(*t*). The subsequent evaluation of the CSD created by this generator allows a comparison to be made with the spatial distributions of the currents during the specific activation of known pathways (Korovaichuk et al., 2010). The pathway specificity of some ICA-isolated components is assessed by their selective capturing of subthreshold-evoked synaptic currents of specific populations or axon bundles (Korovaichuk et al., 2010) and by selective cross-correlation of identified presynaptic units with the temporal envelope of the separated component (Fernández-Ruiz et al., 2012).

The time evolution of the power of an LFP-generator is given by (measured in mV^2^): *P*(*t*) = ∫*H*(*t*−τ)*v*^2^(τ)*d*τ, where *v*(*t*) is the virtual LFP at the electrode with maximal power and *H* is the appropriately scaled square kernel of the length Δ. The mean power is then defined for Δ extended to the complete time interval (about ten min in our experiments).

CSD analysis (Freeman and Nicholson, 1975) determines the magnitude and location of the net transmembrane current generated by neuronal elements contained within a small region of tissue. Accordingly, we used the one-dimensional approach, which calculates the CSD from the voltage and conductivity gradients along the cells axis. This approach requires homogeneous activation of the recorded neuronal population, which is commonly accepted for evoked potentials in the hippocampus (Herreras, 1990). While this may not hold for ongoing LFPs whose current generators may be spatially restricted, it has been shown not to be the case for Schaffer LFP-generator (Fernández-Ruiz et al., 2012), since the Schaffer collaterals produce homogeneous activation in the XY-plane, whether spontaneous or synchronous activity. Thus, for the current purposes the ongoing Schaffer-specific activity is coherent enough as to validate the use of the unidimensional approach for CSD estimation.

### Retrieval and quantification of micro-field excitatory postsynaptic potentials

The baseline activity of Schaffer-LFPs is composed of regular succession of small field potential wavelets at gamma-frequency (Figure 1F). Each of these wavelets was previously shown to correspond to an excitatory package elicited in the CA1 pyramidal cell population by synchronous presynaptic firing of a group of CA3 pyramidal cells or functional cluster, the so called micro-field excitatory postsynaptic potentials (μ-fEPSPs) (Fernández-Ruiz et al., 2012). To study the features of ongoing μ-fEPSPs we measured them as follows.

Let *v(t)* be the Schaffer-specific LFP at the electrode with maximal power (see e.g., Figure 1D). To identify elementary μ-fEPSPs we used the Wavelet Transform of *v(t)*:

$$W(a,b)=\frac{1}{\sqrt{a}}\int v(t)\psi \left(\frac{t-b}{a}\right)dt$$

where ψ is the Haar mother wavelet (well suited for detection of short pulses in a signal), *a* is the time scale and *b* is the localization in time. We then rectified the wavelet coefficients using the following equation:

$$C(a,b)=\frac{1}{\sqrt{a}}\text{max}\left(-W(a,b),0\right)$$

The 2D surface obtained describes the local linear fit of the Schaffer-specific LFP by the pulse-like function (Haar) at the scale *a* and localization *b*. Large absolute values of *C(a,b)* at a given time instant and scale correspond to abrupt pulse-like transitions in *v(t)*. Thus, we can associate such points in the *(b,a)*-plane with singular LFP events. Consequently, the local maxima

$${\left(a,b\right)}_{k}=\text{arg}\underset{\omega_{k}}{\text{max}}(C(a,b))$$

define the time instants of μ-fEPSPs (given *by t*_*k*_ = *b*_*k*_ − *a*_*k*_/2), their duration (given by *a*_*k*_), and amplitudes [given by *A*_*k*_ = *C*(*a*_*k*_,*b*_*k*_)]. It should be noted that the identification of μ-fEPSPs is blind; hence their significant correlation with CA3 or CA1 spikes corroborates the reliability of the technique (see also Fernández-Ruiz et al., 2012).

### Spike sorting, unit classification, and statistical tests

Spike trains of individual units were obtained from unfiltered recordings using wavelet-enhanced spike sorting (Pavlov et al., 2007) and local CSD methods. Units were classified into two subclasses, pyramidal cells and putative interneurons, according to the location of the recording site (within or outside the pyramidal body layer) and additional standard electrophysiological criteria (Csicsvari et al., 1998): (1) spike width (>0.4 ms and <0.4 ms for pyramids and putative interneurons, respectively); (2) mean firing rate (<5 Hz *vs.* >5 Hz); (3) relation to theta rhythm (firing rate decreases for pyramidal cells and increases or remains unchanged for interneurons); (4) pattern of firing (isolated spikes *vs.* bursting); (5) presence of complex spikes (in pyramidal cells only); and (6) the decay of autocorrelograms (fast *vs.* slow). The total number of units employed in this study is limited by the use of linear tracks of recording sites required to collect spatial maps of LFPs for ICA. Typically, 1–3 CA3 and 1-2 CA1 pyramidal cells were successfully isolated per recording.

STAs of CA1 LFPs were obtained from spikes series of single CA3 units containing at least 1500 spikes. The level of significance was determined using the surrogate test (1000 trains with randomly shuffled inter-event intervals: α ≤ 0.05). The standard Student's *t*-test was employed to analyze the differences between two sample means. The casual ratio of coincident spikes was estimated analytically assuming a Poisson distribution of the number of events within a given time interval.

### Indices of in-cluster presynaptic firing, schaffer-driven spikes, and spike transfer rate

The availability of ongoing Schaffer-specific μ-fEPSPs enables the study of neuron-to-population ongoing relations locally amongst CA3 neurons and between these and postsynaptic CA1 neurons. Temporal relationships between spikes of pre- or postsynaptic units and μ-fEPSPs were defined as coincidences with appropriated time windows for monosynaptic connection. For simplicity we assume that only postsynaptic spikes time-related to individual Schaffer μ-fEPSPs are initiated by significant input from this pathway. Several indices were defined to quantify three types of coincidences between elements in the synaptic chain.

#### In-cluster presynaptic firing

Spontaneous firing of CA3 cells occurs with a high degree of synchrony within functional clusters or groups of pyramidal cells (Hájos and Paulsen, 2009; Takahashi et al., 2010). In previous work we reported that each μ-fEPSP composing the baseline of Schaffer-LPFs is produced by co-firing of a group of CA3 pyramidal cells (Fernández-Ruiz et al., 2012). Firing of a unit outside the functional cluster does not produce strong enough (readable) μ-fEPSP. To quantify the presynaptic “in-cluster” firings (or Type I coincidences) we introduced the following index:

$$R_{\text{in-clust}}=\frac{N_{\text{CA3},\mu -\text{fEPSP}}}{N_{\text{CA3}}}$$

where *N*_CA3_ is the number of spikes of a CA3 pyramidal neuron and *N*_CA3,μ−fEPSP_ is the number of μ-fEPSP events synchronous (0–8 ms latency) with CA3 firings. This index varies from 0 to 1 and implicitly describes the variability of functional clusters. Thus, when *R*_in−clust_ is low, the neuron rarely participates in clustered firings, whereas values of *R*_in−clust_ closer to 1 indicate that the neuron always fires synchronously with other neurons.

#### Schaffer-driven spikes

Postsynaptic spikes typically have a variable and possibly multisynaptic origin. We sort those spikes produced by CA1 neurons that are exclusively or decisively initiated by only one of the multiples inputs, the Schaffer synaptic input. To quantify the ratio of spikes of CA1 pyramidal neurons causally associated with μ-fEPSP events (or Type II coincidences), we introduced the index of Schaffer-driven CA1 spikes:

$$R_{\text{Sch-driven}}=\frac{N_{\mu -\text{fEPSP},\text{CA}1}}{N_{\text{CA1}}}$$

where *N*_CA1_ is the number of spikes of a CA1 pyramidal neuron and *N*_μ−fEPSP,CA1_ is the number of CA1 spikes synchronous (0–6 ms latency) with μLFP-events. Again this index ranges from 0 to 1, so that a small *R*_Sch−driven_ value suggests that the Schaffer input has no effect on firing of CA1 cell, whereas *R*_Sch−driven_ value close to one would indicate that the output of the CA1 neuron is completely conditioned by the Schaffer input.

#### Spike transfer rate

Spike transfer amongst synaptically connected units is normally examined by cross-correlating spike trains of pre- and postsynaptic units. The multiple synaptic origins of spikes in a train series make these correlations poorly informative since only a fraction of them is fired by input from the examined afferent pathway. The availability of pathway-specific mediating μ-fEPSPs enables narrowing the study by selecting spikes in both sides that are time-locked to excitatory events from a unique presynaptic population (Fernández-Ruiz et al., 2012). It is important to note that the triple correlation implicitly tackles successful spike production in the postsynaptic side, enabling the estimation of cell-to-cell spike transfer rate to be quantified in non-stimulated conditions. Triple coincidences (also termed Type III) thus represent presynaptic CA3 spikes time-locked to μ-fEPSP events, which in turn drive postsynaptic spikes in CA1 cells. We represented these triple correlations in two-dimensional densitograms in which we considered successful monosynaptic coincidences those falling within time window of 6 × 8 ms (or time window *a*). The density of cell-to-cell efficient monosynaptic events was normalized to the density of casual events in a 30 × 30 ms time window *b*

$$R_{\text{Spike-transfer}}=\frac{D_{a}}{D_{b}}$$

A ratio higher than 1.2 (20% growth) was considered indicative of an effective functional monosynaptic connection in the CA3-CA1 neuronal pair. This way of selecting postsynaptic spikes is akin to histograms of firing probability in evoked responses upon Schaffer electrical stimuli: those fired out of the evoked fEPSP time window are excluded since their synaptic trigger is unknown.

### Conflict of interest statement

The authors declare that the research was conducted in the absence of any commercial or financial relationships that could be construed as a potential conflict of interest.
